# Impact of social isolation due to COVID-19 on the seasonality of pediatric respiratory diseases

**DOI:** 10.1371/journal.pone.0243694

**Published:** 2020-12-11

**Authors:** Milena Siciliano Nascimento, Diana Milena Baggio, Linus Pauling Fascina, Cristiane do Prado

**Affiliations:** Department of Pediatrics, Hospital Israelita Albert Einstein, São Paulo, Brazil; University of Cape Town, SOUTH AFRICA

## Abstract

**Introduction:**

Respiratory tract diseases are the major cause of morbidity and mortality in children under the age of 5 years, constituting the highest rate of hospitalization in this age group.

**Objectives:**

To determine the prevalence of hospitalizations for respiratory diseases in childhood in the last 5 years and to assess the impact of social isolation due to COVID-19 on the seasonal behavior of these diseases.

**Methods:**

A cross-sectional clinical study was carried out, with a survey of all patients aged 0 to 17 years who were admitted with a diagnosis of respiratory diseases between January 2015 and July 2020. The database was delivered to the researchers anonymized. The variables used for analysis were date of admission, date of discharge, length of stay, age, sex and diagnosis. In order to make the analysis possible, the diagnoses were grouped into upper respiratory infection (URI), asthma / bronchitis, bronchiolitis and pneumonia.

**Results:**

2236 admissions were included in the study. Children under 5 years old account for 81% of hospitalizations for respiratory disease in our population. In the adjusted model, an average reduction of 38 hospitalizations was observed in the period of social isolation (coefficient: -37.66; 95% CI (- 68.17; -7.15); p = 0.016).

**Conclusion:**

The social isolation measures adopted during the COVID-19 pandemic dramatically interfered with the seasonality of childhood respiratory diseases. This was reflected in the unexpected reduction in the number of hospitalizations in the pediatric population during this period.

## Introduction

Respiratory tract diseases are the major cause of morbidity and mortality in children under the age of 5 years, constituting the highest rate of hospitalization in this age group [1, 2]. In developing countries, diseases of the lower respiratory tract represent about 90% of deaths from respiratory diseases, most of which are bronchial and alveolar infections of viral origin [1, 3]. Regarding upper respiratory infections (URI), infants and preschoolers develop, on average, six to eight infections per year [4]. This infection profile, despite not causing serious illness, is responsible for epidemics due to the continuous circulation of pathogens in the community [5].

In this context, it is important to highlight the role of day-care centers and schools that start in increasingly younger age groups and several studies point out as being an important risk factor for the acquisition of respiratory infections, especially in children from zero to two years of age, due to greater children's exposure to infectious agents, confinement and agglomeration. In addition, children can act as sources of infection in their families, further spreading infectious agents in the community [5, 6].

In the year 2020, due to the pandemic caused by COVID-19, social isolation, with the closing of trade and companies and the suspension of classes, was highly recommended in an attempt to reduce the spread of the virus. The impact caused by social isolation was one of the factors that may have contributed to the change in the seasonal characteristic of respiratory diseases in pediatrics in the year in question [7].

In Brazil, social isolation measures that were included suspending classes at schools and universities, closing companies and commerce and banning events, have been started in mid-March. The easing of these measures began in mid-July, however, schools and day care remain closed [8].

The present study aims to determine the incidence of hospitalizations for respiratory diseases in childhood in the last 5 years and to assess the impact of social isolation due to COVID-19 on the seasonal behavior of these diseases.

## Methods

### Study type and location

A cross-sectional study was carried out by collecting epidemiological data on hospital admissions for respiratory diseases in pediatric patients, at private hospital from January 2015 to July 2020. Patients aged 0 to 17 years were included, who needed of hospitalization with diagnoses of respiratory diseases. Our institution is a tertiary level private hospital that providence approximately 3,000 admissions per year

### Protocol

After approval of the project by the Research Ethics Committee of the Hospital Israelita Albert Einstein, a survey was made of all patients aged 0 to 17 years who were hospitalized with primary and secondary diagnosis of respiratory diseases (International Disease Code, 10th Revision: J00 –J99) from January 2015 to July 2020. The database was delivered to the researchers anonymously and, for this reason, the informed consent term was waived by the ethics and research committee.

The variables used for analysis were date of admission, date of discharge, length of stay, age, sex and diagnosis. To make the analysis possible, the diagnoses were grouped using the subcategories by similarity criteria into upper airway infection (URI) asthma / bronchitis, bronchiolitis and pneumonia. The exact grouping of the ICD-10 codes into each subcategory was specified in Table 1. Asthma and bronchitis were grouped because diagnosis of bronchitis was closely associated with the secondary diagnosis of asthma.

**Table 1:** ICD-10 codes into each subcategory.

Upper airway infection (UAI)	J02.8 Acute pharyngitis due to other specified organisms
	J02.9 Acute pharyngitis, unspecified
	J04.0 Acute laryngitis
	J04.1 Acute tracheitis
	J04.2 Acute laryngotracheitis
	J06.9 Acute upper respiratory infection, unspecified
	J38.6 Stenosis of larynx
	J39.2 Other diseases of pharynx
Asthma /Bronchitis	J45.1 Nonallergic asthma
	J45.8 Mixed asthma
	J45.9 Other and unspecified asthma
	J20.0 Acute bronchitis due to Mycoplasma pneumoniae
	J20.5 Acute bronchitis due to respiratory syncytial virus
	J20.6 Acute bronchitis due to rhinovirus
	J20.8 Acute bronchitis due to other specified organisms
	J20.9 Acute bronchitis, unspecified
	J21.9 Acute bronchiolitis, unspecified
	J40 Bronchitis, not specified as acute or chronic
Bronchiolitis	J21.0 Acute bronchiolitis due to respiratory syncytial virus
	J21.8 Acute bronchiolitis due to other specified organisms
Pneumonia	J10.1 Influenza due to other identified influenza virus with other respiratory manifestations
	J12.0 Adenoviral pneumonia
	J12.1 Respiratory syncytial virus pneumonia
	J12.2 Parainfluenza virus pneumonia
	J12.8 Other viral pneumonia
	J15.7 Pneumonia due to Mycoplasma pneumoniae
	J15.8 Pneumonia due to other specified bacteria
	J15.9 Unspecified bacterial pneumonia
	J17 Pneumonia in diseases classified elsewhere
	J18.0 Bronchopneumonia, unspecified organism
	J18.9 Pneumonia, unspecified organism

### Statistical analysis

The data were described by means of absolute and relative frequencies for categorical variables and by mean values and standard deviations (SD), minimum, maximum and quartiles for numerical variables. The distributions of continuous variables were investigated using boxplots and histograms.

Generalized linear models with Gamma distribution and logarithmic link function were adjusted to estimate the length of hospital stay and to investigate associations with periods of social isolation Categorical variables were compared between groups with or without social isolation due to COVID-19 using chi-square or Fisher’s exact tests

The number of hospitalizations over time and their relationship with social isolation was assessed by a time series regression model, with ARIMA identification. The model considered the monthly numbers of hospitalizations for respiratory diseases in pediatric patients in the first semester and social isolation in the period from April to June 2020 was used as an explanatory variable, with the coefficient and p-value for the absence of alteration test being presented. and the level of significance adopted was 5%.

The analyzes were performed with the aid of the SPSS program (version 24.0) and the R (version 4.0.2) and the additional packages: forecast, Rcmdr, RcmdrPlugin.EZR and lmtest. For all analyzes, the 5% significance level was adopted.

## Results

The database contained 2618 records and hospitalizations, of which 382 were excluded, as they had primary and secondary diagnoses that did not meet the study's inclusion criteria. Thus, 2236 admissions were included in the study.

The main demographic characteristics as well as the presentation of the main diagnoses are shown in Table 2.

**Table 2:** Characteristics of pediatric patients admitted for respiratory diseases in the period from January 2015 to June 2020 (n = 2236).

	Total (2236)	Social isolation due to COVID-19		p-value
		No (n = 2216)	Yes (n = 20)	
Sex				0.571 ^1^
Female	1089 (48.7%)	1078 (48.6%)	11 (55.0%)	
Male	1147 (51.3%)	1138 (51.4%)	9 (45.0%)	
Age group				0.002 ^2^
0 to 2 years old	1267 (56.7%)	1260 (56.9%)	7 (35.0%)	
3 to 5 years old	543 (24.3%)	541 (24.4%)	2 (10.0%)	
6 to 10 years old	331 (14.8%)	322 (14.5%)	9 (45.0%)	
11 to 17 years old	95 (4.2%)	93 (4.2%)	2 (10.0%)	
Diagnosis based on CID*				0.153 ^2^
Asthma / Bronchitis	223 (10.0%)	221 (10.0%)	2 (10.5%)	
Bronchiolitis	163 (7.2%)	163 (7.3%)	0 (0.0%)	
UAI (upper airway infection)	249 (11.1%)	244 (11.0%)	5 (26.3%)	
Pneumonia	1601 (71.6%)	1589 (71.7%)	13 (63.2%)	
Infection Agent				0.141 ^1^
Bacterium	1466 (65.6%)	1456 (65.7%)	10 (50.0%)	
Virus	770 (34.4%)	760 (34.3%)	10 (50.0%)	
Length of hospital stay (days) ^#^				
Mean (IC 95%)	4.21 (4.09; 4.34)	4.23 (4.10; 4.35)	2.70 (1.98; 3.69)	0.005 ^3^
Minimum; Maximum	0.13; 112.00	0.13; 112.00	1.00; 6.00	

Without social isolation: january/2015 –march/2020; with social isolation: april/2020 –june/2020

p-values for the chi-square test(1), Fisher’s exact test(2) and generalized linear model (3).

#: mean and 95% confidence interval estimated by generalized linear model. For those with less than one day in the hospital, we assumed three hours of stay, which equates to 0,125 days of hospitalization

The main demographic characteristics as well as the presentation of the main diagnoses are shown in Table 1. This table also shows the comparisons between period with (April/2020 –June/2020) or without social isolation (January/2015 –march/2020). Children under 5 years old accounted for 81.3% of hospitalizations for respiratory disease in the period without social isolation. During social isolation we observed a significant reduction, with children under 5 years old representing 45% of hospitalizations (p-value = 0.002). We also observed a reduction in the length of hospital stay (p-value = 0,005).

**Fig 1:**
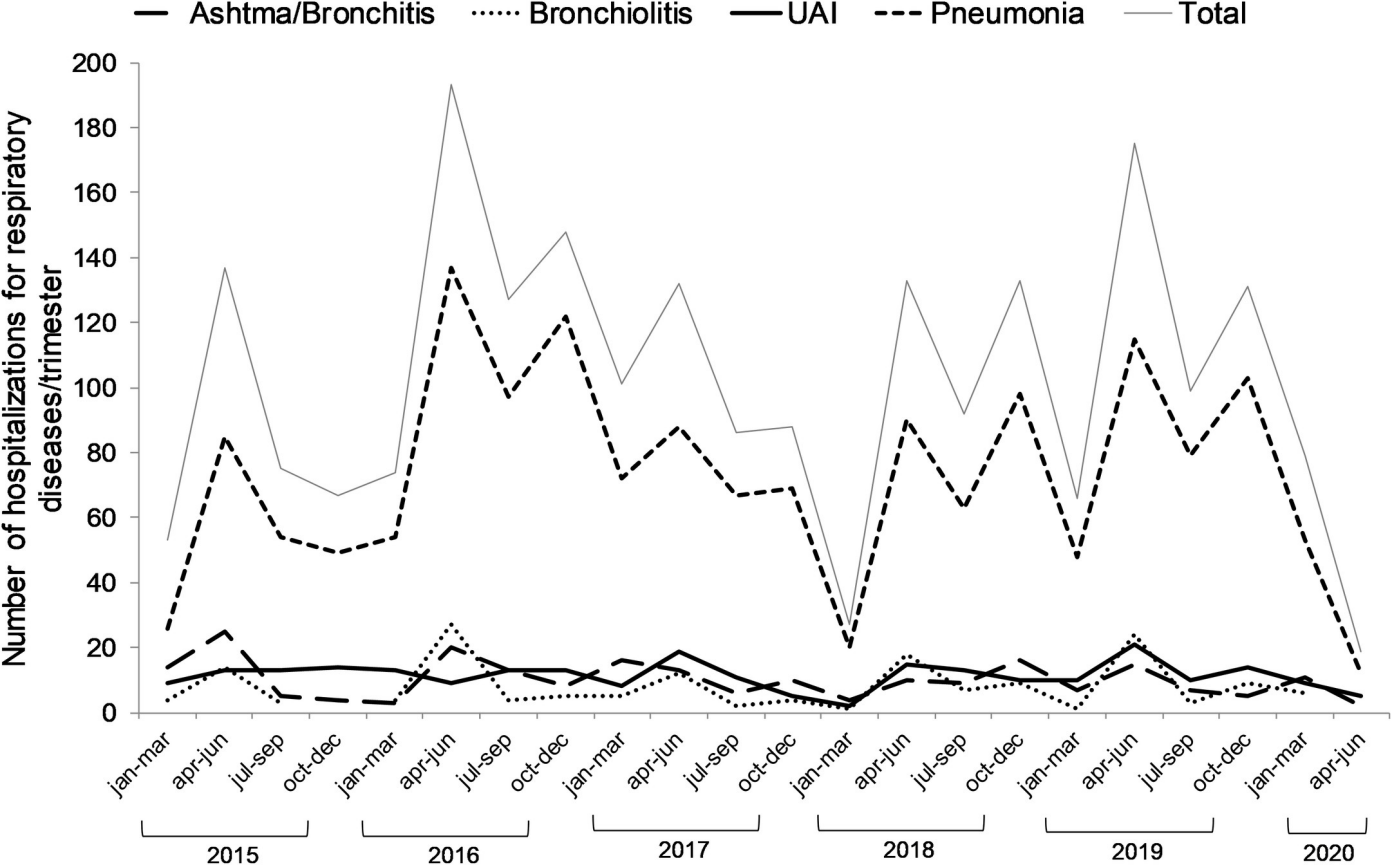
Distribution of the number of hospitalizations for respiratory diseases in pediatric patients from January 2015 to June 2020 total and according to diagnosis (n = 2236).

The distribution of the number of hospitalizations per trimester in the period from 2015 to 2020, total and according to diagnosis is shown in Fig 1.

In the ARIMA model, we observed in the period with social isolation (April to June 2020) an average reduction of 38 hospitalizations for respiratory diseases in pediatric patients (coefficient: -37.66; 95% confidence interval: -68.17 to -7, 15; *p* = 0.016).

**Fig 2:**
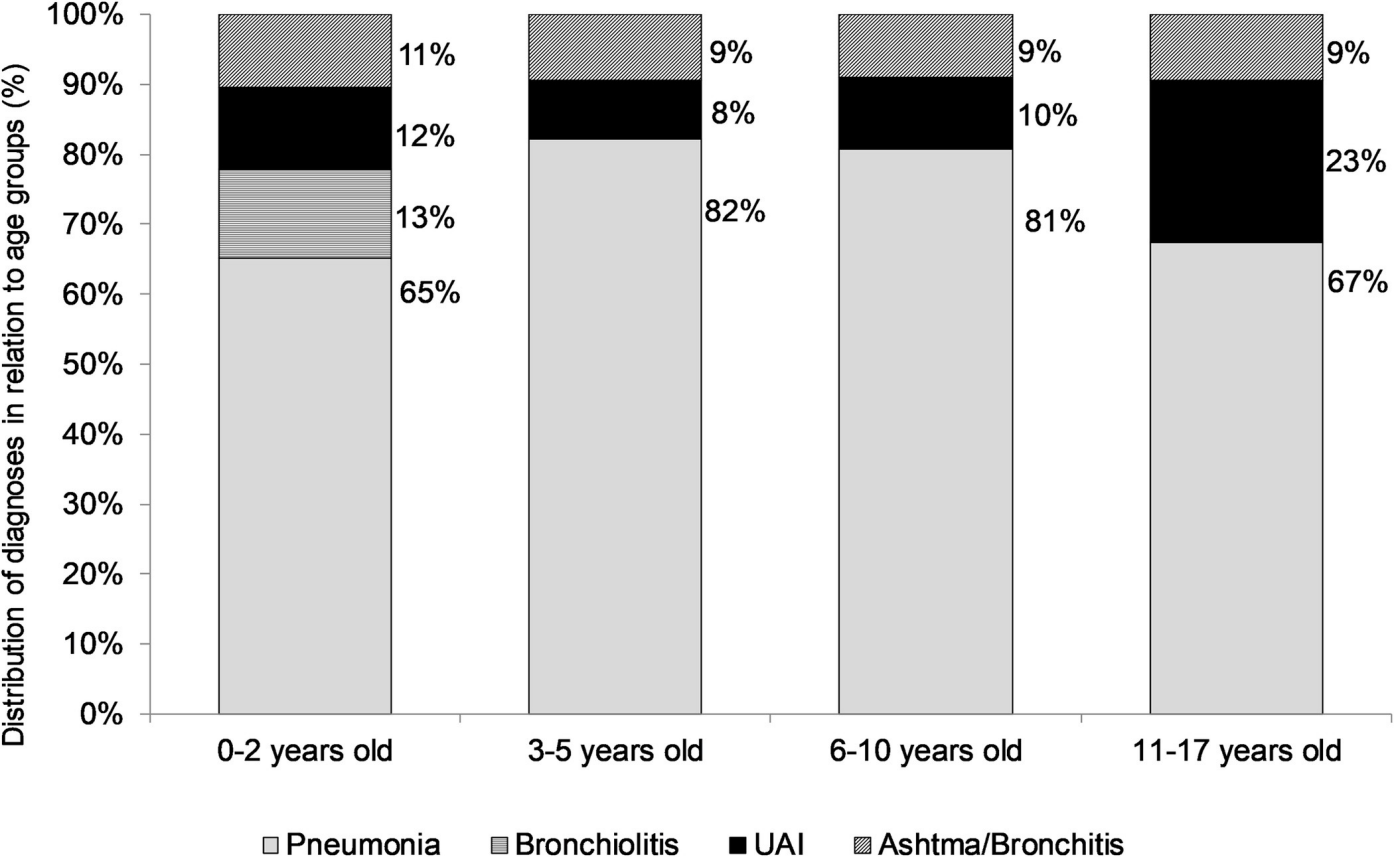
Inpatient diagnoses by age group of pediatric patients admitted for respiratory diseases in the period from January 2015 to June 2020.

The distribution behavior of the total number of admissions and hospitalizations diagnosed with pneumonia is quite similar given the proportion of pneumonia cases (over 60%). This predominance of pneumonia cases can also be seen in Fig 2 that shows the distribution of inpatient diagnoses by age group.

Evidence of association of the age group with the diagnosis of hospitalization was found (*p* <0.001) (Table 3), with highlights for greater proportions of cases of pneumonia in the age groups from three years old than in the age group of zero to two years and all cases bronchiolitis in the range up to two years of age. In the multiple comparisons, no difference was found between the groups of 3 to 5 years old and 11 to 17 years old.

**Table 3:** Relationship between diagnosis and age groups (n = 2236).

Diagnosis	Age groups			
	0 to 2 years old	3 to 5 years old	6 to 10 years old	11 to 17 years old
Ashtma/Bronchitis	133 (10.5%)	51 (9.4%)	30 (9.1%)	9 (9.5%)
Bronchiolitis	163 (12.8%)	0 (0.0%)	0 (0.0%)	0 (0.0%)
UAI	147 (11.6%)	46 (8.5%)	34 (10.3%)	22 (23.2%)
Pneumonia	824 (65.1%)	446 (82.1%)	267 (80.7%)	64 (67.4%)
Total	1267 (100.0%)	543 (100.0%)	331 (100.0%)	95 (100.0%)
Global chi-square p-value	<0.001 ^1^			
Multiple comparisons Bonferroni corrected p values				
versus 0 to 2 years old		<0.001 ^2^	<0.001 ^2^	<0.001 ^2^
versus 3 to 5 years old			>0.99 ^2^	0.002 ^2^
versus 6 to 10 years old				0.033 ^2^

p-values for the chi-square test ^(1)^ and Fisher’s exact test ^(2)^

**Fig 3:**
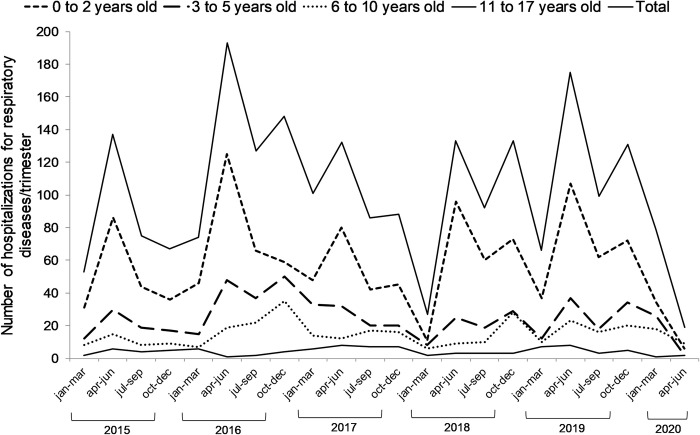
Distribution of the number of hospitalizations for respiratory diseases in pediatric patients from January 2015 to June 2020, according age group.

In Fig 3, we see the distribution of the number of hospitalizations per month in the period from 2015 to 2020 for each age group in which the predominance of the age group from a to 2 years is observed very clearly, mainly in the months of seasonal peaks.

## Discussion

Our study describes the epidemiological profile of hospitalizations for respiratory diseases in the last 5 years and brings evidence that social isolation has significantly reduced hospitalizations for respiratory diseases in the pediatric population.

Hospitalizations for respiratory diseases in children and adolescents usually present a distribution pattern dependent on the age group [1, 2] and seasonality [9].

In our study, children under 5 years old represented 81% of hospitalizations and pneumonia were the main diagnoses (over 65%) in all age groups, however, with a higher proportion of pneumonia cases in the age group from three years. Respiratory diseases of viral etiologies (infections of upper airways, bronchiolitis) are cited as being more common in the pediatric population [9, 10], which contradicts our founds. However, other studies corroborate our results and point to pneumonia as a diagnosis in more than 50% of all hospitalizations for respiratory diseases [2, 11, 12].

In our study, when comparing the period with isolation and without isolation, we observed a significant increase in the percentage of hospitalizations in the group of 6 to 10 years. Kuitunen I et al found no difference in the age distribution of patients in periods with and without isolation [13]. We believe that this percentage increase in the number of hospitalizations in the 6 to 10 age group is related to the drastic reduction in the number of hospitalizations during the isolation period. During this period, only 20 patients required hospitalization for respiratory diseases.

Several studies confirm the occurrence of a seasonal peak in respiratory diseases and point to an association with environmental factors such as tobacco exposure, humidity, temperature and an increase in the level of pollutants [14–16]. Sudden changes in temperatures associated with the worsening of the quality of the inspired air are contributing factors for a significant increase in cases of pneumonia, asthma and bronchiolitis [17, 18]. Our results also demonstrated this seasonality pattern of respiratory diseases in the period from April to June 2015 to 2019, the exception being the year 2020.

Other factors such as the presence of siblings and crowding are also associated with an increased risk of hospitalization for respiratory diseases [12]. Attendance to daycare centers and schools are also identified as contributing factors, not only in the increase in the incidence of respiratory diseases in childhood, but also in the maintenance of the circulation of pathogens [19, 20].

The pandemic caused by COVID-19 in 2020 and the significant reduction in the number of hospitalizations for respiratory diseases in the pediatric population observed by our study demonstrates that, agglomerations and attendance at daycare centers and schools, have a greater contribution share than if could study, as they are difficult to control variables in routine situations.

Another 2 recently published studies confirm our findings and strongly suggest that social distancing and other lockdown strategies are effective to slow down the spreading of respiratory diseases and decreasing the need for hospitalization among children [13, 21].

Since then, the exponential growth in the number of cases, resulting in the collapse of the health system in different countries, has led several governments to adopt control measures to reduce the levels of transmission [22, 23]. These measures included suspending classes at schools and universities, closing companies and commerce and banning events. A study that evaluated the effectiveness of adopting such measures in slowing the growth rates of COVID-19 cases demonstrated that this has a direct association with the time of the epidemic in which they were adopted. In Italy and Spain, control measures were taken at the national level in a final stage of the epidemic, which may have contributed to the high spread of COVID-19 in these countries. In Brazil, the measures adopted prevented the collapse of the health system and appear to have an influence on the growth curve of new infections by COVID-19 [7, 8].

In addition to social isolation measures, the education of the population in relation to the use of masks and hand hygiene were also factors that contributed to curb the circulation of pathogens. Rare were cases of bronchiolitis, highly prevalent diseases at this time of year [24, 25].

Historically, since the Spanish flu, we have not had this spectrum of social isolation including closing schools and daycare centers. This fact transformed the seasonality model of pediatric respiratory diseases. Currently, we only have the question: What will 2021 be like?

## Conclusion

The social isolation measures adopted during the COVID-19 pandemic dramatically interfered with the seasonality of childhood respiratory diseases. This was reflected in the unexpected reduction in the number of hospitalizations in the pediatric population during this period.

## Supporting information

S1 File (XLSX)
