# Supplementary material for: Impact of social isolation due to COVID-19 on the seasonality of pediatric respiratory diseases
Source: PLoS One. 2020 Dec 11;15(12):e0243694. doi: 10.1371/journal.pone.0243694 (PMC7732104; doi:10.1371/journal.pone.0243694)
Supplement: Attachment — Submitted filename: reviewer comments PLOS ONE.pdf [file pone.0243694.s002.pdf]

PLOS ONE manuscript review

Impact of social isolation due to COVID-19 on the seasonality of pediatric respiratory diseases

This is an important paper that highlights the effect of public health strategies instituted to control the spread of COVID-19 globally in 2020. It highlights that these strategies yielded an overall reduction in hospitalisations due to lower respiratory tract infections especially in the under 5 age groups.

My comments below for the authors to consider:-

Abstract:

Rephrase the sentence….. we will only know if there is a change in behaviour observed in 2020, it will also influence the seasonality of 2021 with the continuity of results for……

Methods

Line 94: Patients aged 0 to 17 years and 11 months….. On the results sections, the highest age recruited was 17 years; probably delete the 11 months part on the methodology or just state children under 18 years were recruited.

Line 103 and 10: regarding groupings of the various diagnosis, how was this classification arrived at? Please see my comment below regarding this classification in Table 1.

Results

Table 1:” to” is missing in the rows reading age group

Age group:

0 a 2 years old

3 a 5 years old

6 a 10 years old

11 a 17 years old

Diagnosis

I am not clear why the diagnosis of pneumonia is separate from Bronchopneumonia instead of classifying these two categories under pneumonia.

Also the diagnosis of bronchitis versus bronchiolitis, how were the two diagnoses differentiated especially in the younger children age-group?

It is unclear in the results section the various viral aetiologies depicted under viral pneumonia yet we see this on line 202 in the discussion.

The row that reads …Tempo de internação (dias).. please write this in English

Line 137-that starts…..In the adjusted model,….A table showing the results of this analysis will be useful.

Figure 1: This trend is for all groups. Possible to show the trend for different age groups in Figure1?

Figure 2: Classify pneumonia and Bronchopneumonia together (please see my comments above). Also make the markings on the graph clearer.

Figure 3: see my comments above on the classification of the various diagnosis. Please make the labels of figure 3 clear. Also rephrase line 147 to 149 which is the narrative for figure 3

Discussion

Line 165- again pneumonia and bronchopneumonia terminologies… Please see my comments above regarding these two terminologies

Conclusion: This is well written and summarises the overall take-home message well from this study
